# C-954-11. Language of Survey Completion and Patient-reported Outcomes Following Burn Injury: English vs. Spanish

**DOI:** 10.1093/jbcr/irag033.143

**Published:** 2026-04-06

**Authors:** Sarah Wang, Amila Adili, Trevor A Pickering, Carly Marincasiu, Kimberly Roaten, Haig A Yenikomshian

**Affiliations:** Keck School of Medicine, University of Southern California, Los Angeles, CA; Southern California Clinical and Translational Science Institute (SC-CTSI), Keck School of Medicine, University of Southern California, Los Angeles, CA; Department of Population and Public Health Sciences, Keck School of Medicine, University of Southern California, Los Angeles, CA; Department of Surgery, University of Washington Medicine Regional Burn Center, Seattle, WA; Department of Psychiatry, University of Texas Southwestern Medical Center, Dallas, TX; Division of Plastic and Reconstructive Surgery, Keck School of Medicine, University of Southern California, Los Angeles, CA

## Abstract

**Introduction:**

Cultural and linguistic factors are important but often under-studied components of socioeconomic status that can influence recovery after a burn injury. For burn survivors, differences in primary language may reflect unique cultural experiences and varying health literacy that impacts navigation of healthcare and recovery. The aim of this study is to examine how language is associated with patient reported outcomes after burn injury and to identify differences in patterns of healthcare utilization.

**Methods:**

A multi-center longitudinal burn database was used to analyze retrospective data of adult burn patients from 2015 - 2024. Participants were categorized based on the language of survey completion (English or Spanish). Patient reported outcome measures for life satisfaction, social outcomes, and medication usage were collected at 12 months post-injury. The association between language and outcomes was modeled by multivariable regression, which adjusted for confounding variables such as demographics (age, sex, race, education) and burn injury characteristics (%TBSA, inhalation injury).

**Results:**

A total of 1106 individuals were included in analyses, and the majority of surveys (n = 960, 86.8%) were completed in English. The English cohort reported significantly worse outcomes in several domains compared to those who completed surveys in Spanish. English-speakers reported lower BSHS Body Image scores (β = -0.49, *p*=.013) and lower Satisfaction with Life scores (β = -8.69, *p*<.001). Patients who completed surveys in English endorsed higher PROMIS pain scores (β = 0.94, *p*=.013) and had lower odds of using pain medications (OR = 0.19, *p*=.004). This cohort also had significantly increased odds of using depression medications (OR = 2.96, *p*=.010).

**Conclusions:**

Following a burn injury, English-speaking patients endorsed worse pain, body image, and life satisfaction compared to Spanish-speaking patients. Further research is needed to determine the etiology of the observed discrepancies in patient reported outcomes; these may reflect variation in psychosocial well-being between linguistic groups related to factors like social cohesion and family support. Cultural factors may also drive differences in the acknowledgement and reporting of mental health challenges and pain.

**Applicability of Research to Practice:**

There is a need for providers to engage in culturally competent care, including the use of certified medical interpreters and culturally sensitive psychological support to address the unique recovery challenges of different linguistic groups.

**Funding for the study:**

The contents of this abstract were developed under a grant from the National Institute on Disability, Independent Living, and Rehabilitation Research (NIDILRR grant number 90DPBU0007) and from the National Center for Advancing Translational Science (NCATS grant number UL1TR001855). NIDILRR is a Center within the Administration for Community Living (ACL), Department of health and Human Services (HHS). The contents of this abstract do not necessarily represent the policy of NIDILRR, ACL, HHS, or NIH and you should not assume endorsement by the Federal Government.

